# 1. ENGAGEMENT: A PRIORITY NEED IN CARE TRANSITIONS EXPERIENCED BY OLDER ADULTS AND CAREGIVERS

**DOI:** 10.1093/geroni/igae098.3771

**Published:** 2024-12-31

**Authors:** Claire Regan, Karen Hirschman, Rachel Harding, Molly McHugh, Onome Osokpo, Kathleen McCauley, Elizabeth Shaid, Mary Naylor

**Affiliations:** University of Pennsylvania, Philadelphia, Pennsylvania, United States; University of Pennsylvania, Philadelphia, Pennsylvania, United States; University of Pennsylvania, Philadelphia, Pennsylvania, United States; University of Pennsylvania, Philadelphia, Pennsylvania, United States; University of Illinois Chicago, Chicago, Illinois, United States; University of Pennsylvania, Philadelphia, Pennsylvania, United States; Penn Nursing, Merion Station, Pennsylvania, United States; University of Pennsylvania, Philadelphia, Pennsylvania, United States

## Abstract

Care transitions are a vulnerable time for older adults, particularly transitions from hospital to home. The Transitional Care Model (TCM) is designed to support older adults and their informal caregivers during this period, consistently demonstrating improvements in health outcomes while lowering care costs. This multi-component, advanced practice registered nurse (APRN)-led, team-based care intervention was recently implemented in a large-scale RCT in three U.S. healthcare systems (9/2020-3/2023). Content analysis of APRN intervention documentation for 480 patients revealed key barriers to delivering the TCM as designed. The lack of patient and/or informal caregiver engagement theme was the most common documented challenge (231/480, 48.1%), higher than issues of complexity of care (166/480, 34.6%) and social determinants of health (SDOH, 138/480, 22.8%). The lack of engagement theme was most often identified as a patient issue (197/231, 85.3%) or patient and caregiver issue (23/231, 10.0%). Subcategories included limited participation in care, patient refusal of services, and lack of adherence to plan of care (e.g. lifestyle changes, medication management and motivation). Lack of engagement was complicated by the complexity of care and/or SDOH (101/231, 43.7%). These findings demonstrate the challenges of patient engagement in the presence of complexity of care and SDOH within transitional care and how difficult engagement of patients and caregivers can be to achieve. Future research is needed to understand the factors contributing to and mediating lack of engagement as well as this challenge’s impact on health outcomes. Addressing this knowledge gap will inform the design of more effective transitional care interventions.

